# Classification of and individual treatment strategies for complex tethered cord syndrome

**DOI:** 10.3389/fsurg.2024.1277322

**Published:** 2024-01-23

**Authors:** Hepu Lin, Hui Su, Cuicui Li, Pengfei Zhang, Bo Xiu, Yunjing Bai, Ruxiang Xu

**Affiliations:** ^1^ Department of Neurosurgery, The First Medical Center of the PLA General Hospital, Beijing, China; ^2^ Department of Neurosurgery, The Seventh Medical Center of the PLA General Hospital, Beijing, China; ^3^ Department of Neurosurgery, Sichuan Provincial People’s Hospital, Chengdu, China

**Keywords:** complex tethered cord syndrome, classification, individual treatment, tethercausing factor, release, decompression

## Abstract

**Objective:**

To study the classification, diagnosis, and treatment strategies of complex tethered cord syndrome (C-TCS) on the basis of the patients’ clinical symptoms, imaging findings, and therapeutic schedule.

**Methods:**

The clinical data of 126 patients with C-TCS admitted to our department from January 2015 to December 2020 were retrospectively analyzed. Classification criteria for C-TCS were established by analyzing the causes of C-TCS. Different surgical strategies were adopted for different types of C-TCS. The Kirollos grading, visual analogue scale (VAS), critical muscle strength, and Japanese Orthopaedic Association (JOA) scores were used to evaluate the surgical outcomes and explore individualized diagnosis and treatment strategies for C-TCS.

**Results:**

C-TCS was usually attributable to three or more types of tether-causing factors. The disease mechanisms could be categorized as pathological thickening and lipomatosis of the filum terminal (filum terminal type), arachnoid adhesion (arachnoid type), spina bifida with lipomyelomeningocele/meningocele (cele type), spinal lipoma (lipoma type), spinal deformity (bone type), and diastomyelia malformation (diastomyelia type). Patients with different subtypes showed complex and varied symptoms and required individualized treatment strategies.

**Conclusion:**

Since C-TCS is attributable to different tether-related factors, C-TCS classification can guide individualized surgical treatment strategies to ensure complete release of the tethered cord and reduce surgical complications.

## 1 Introduction

Tethered cord syndrome (TCS) is a common disease characterized by developmental malformations of the spine and spinal cord (1, 2). Most of the cases of TCS are congenital while a few are acquired. In this study, all of the patients showed congenital developmental malformations and were newly diagnosed patients. The imaging manifestations of this disease are varied, and mainly include a low spinal cord, myelolipoma, lipomyelomeningocele/meningocele, syringomyelia, diastemastomyelitis, spina bifida, scoliosis, and fur sinuses (3–6). Complex TCS (C-TCS) is accompanied by complex and varied spinal cord manifestations and nerve root adherents along with severe lumbosacral coccygeal vertebrae deformities (7). Patients with C-TCS may also show excessive lordosis or kyphosis of the spine, torsion deformation, severe spinal canal stenosis, and other such changes that may make it almost impossible to distinguish the normal anatomical structure (8). The severe clinical symptoms, complex imaging manifestations, and disorganized anatomical structure increase the difficulty of surgical treatment (9, 10). This study retrospectively analyzed the clinical data of patients with C-TCS admitted to the Neurosurgery Department of the Seventh Medical Center of the People's Liberation Army General Hospital from January 2015 to December 2020. The clinical symptoms, imaging findings, treatment methods and other clinical characteristics of these patients were studied, and the classification and diagnosis and treatment strategies of C-TCS were summarized.

## 2 Patients and methods

### 2.1 General information

A total of 126 patients (46 males, 80 females; age, 3 months–65 years; mean age, 23 ± 2.3 years) with C-TCS were included. The study cohort included 103 cases of lower-extremity dysfunction, 109 cases of urine and bowel abnormalities, 45 cases of sexual dysfunction, 122 cases of skin abnormalities, and 37 cases of spinal deformity (Table 1). We included patients showing (1) presence of congenital TCS; (2) more than three types of thrombolytic factors; and (3) worsening of symptoms in the last three years. On the other hand, we excluded (1) patients with acquired TCS; (2) patients who had undergone tethered cord-related surgery; (3) patients with two or less thrombogenic factors; and (4) patients with incomplete clinical data. These patients were divided into four groups: A, B, C and D which respectively included three, four, five, and six tether-causing factors.

**Table 1:** Basic information of the patients.

Total number of patients	126
Male	46
Female	80
Age	3 months–65 years
Mean age	23 ± 2.3 years
Lower-extremity dysfunction	103
Urine and bowel abnormalities	109
Sexual dysfunction	45
Skin abnormalities	122
Spinal deformity	37

### 2.2 Clinical signs and symptoms

The main symptoms were urinary and bowel dysfunction, lower limb dysfunction, and sexual dysfunction, consistent with the theory that higher-level nerves were less damaged while lower-level nerves were inevitably damaged in this disease. The specific manifestations included frequent urination, urgent urination, weak urination, incomplete dripping, dysuria, urinary retention, urinary incontinence, dry stool, irregular defecation, difficulty defecation, fecal incontinence, paresthesia such as pain and numbness of both lower limbs, weakness of both lower limbs, bipedal deformity, muscle atrophy and even paralysis of lower limbs, and erectile dysfunction. The main signs included the presence of lumbosacral skin masses, meat tags or skin depressions, local skin pigmentation, abnormal hair distribution, fur sinus, scoliosis, or even kyphosis or lordosis. According to JOA score, the severity of clinical manifestations of the patients was divided into mild (25 cases): 25–29 points, moderate (48 cases): 16–24 points, severe (36 cases): 10–15 points, extremely severe (17 cases): <10 points, and the distribution of the severity of clinical manifestations in the 4 groups were listed (Table 2). All patients showed varying degrees of symptom aggravation within 3 years.

**Table 2:** Distribution of severity of clinical manifestations in 4 groups.

	A group	B group	C group	D group	Total
Mild	18 (25.4%)	6 (16.2%)	1 (9.1%)	0	25 (19.8%)
Moderate	32 (45.1%)	12 (32.4%)	3 (27.3%)	1 (14.4%)	48 (38.1%)
Severe	15 (21.1%)	15 (40.6%)	3 (27.3%)	3 (42.8%)	36 (28.6%)
Extremely severe	6 (8.4%)	4 (10.8%)	4 (36.3%)	3 (42.8%)	17 (13.5%)
Total	71	37	11	7	126

In general, the more tether-causing factor, the more severe the clinical manifestations. There were more mild and moderate cases in group A, more moderate and severe cases in group B, more severe and extremely severe cases in group C and D, and even no mild cases in group D.

### 2.3 Imaging manifestations

Lumbosacral vertebral MRI was performed in all patients. The main imaging manifestations included a low spinal cord, myelolipoma, lipomyelomeningocele (meningocele), syringomyelia, diastemastomyelitis, spina bifida, and sacral cysts in a few patients. These common imaging abnormalities indicate that C-TCS is associated with a severe spinal cord end and nerve root adherents and significant lumbosacral coccygeal deformities, excessive lordosis, kyphosis, torsion deformation or severe spinal canal stenosis, which make it almost impossible to distinguish the normal anatomical structure. CT scans revealed the absence of lumbosacral spinous processes and lamina, formation of bone spurs within the spinal canal, abnormal free bone, spinal canal stenosis or pathological dilatation, lumbosacral coccygeal lordosis, kyphosis, lateral curvature, or torsion.

### 2.4 Other examinations

All patients underwent bladder residual urine ultrasonography, electromyography of both lower limbs, and urodynamic examinations to provide objective examination data for evaluating the status of patients before and after the operation.

### 2.5 Surgical treatment

The main causes of C-TCS include pathological thickening and lipomatosis of the filum terminal (filum terminal type), arachnoid adhesion (arachnoid type), spina bifida with lipomyelomeningocele/meningocele (cele type), spinal lipoma (lipoma type), spinal deformity (bone type), and diastomyelia malformation (diastomyelia type). C-TCS is usually caused by three or more thrombolytic factors. The tether-causing factors differ among patients, and the imaging findings are complex and varied. Therefore, surgical strategies should differ according to the tether-causing factors; thus, each patient requires individualized surgical treatment strategies.

### 2.6 Follow-up and evaluation

The Kirollos scale, visual analogue scale (VAS), muscle strength, and Japanese Orthopaedic Association (JOA) scores were used to assess the postoperative improvement in clinical symptoms and thereby evaluate the surgical effect. The follow-up period was 12–60 months. Lumbosacral vertebral MRI, residual urine ultrasound of the bladder, electromyography of both lower limbs, and urodynamics examination were performed in the follow-up assessments.

## 3 Results

### 3.1 Diagnosis and treatment strategy and surgical effect

Pathological thickening and lipomatosis of the filum terminal (filum terminal type), arachnoid adhesion (arachnoid type), spina bifida with lipomyelomeningocele/meningocele (cele type), spinal lipoma (lipoma type), spinal deformity (bone type), and diastomyelia malformation (diastomyelia type) are common tether-causing factors in TCS. However, C-TCS often manifests with three or more of these tether-causing factors, complicating the disease and greatly increasing the difficulty of surgery. The number of times which the filum terminal, arachnoid, cele, lipoma, bone, and diastomyelia types, appeared respectively was 120, 112, 78, 65, 34, and 27 (Table 3). The statistical data indicated that almost all patients showed the filum terminal-type and arachnoid-type tether-causing factors, and that C-TCS may especially present with more than four types of tether-causing factors. The greater the number of tether-causing factors, the more difficult the operation, and the presence of the bone and diastomyelia types is associated with an especially difficult operation. Individual diagnosis and treatment strategies for different tether-causing factors are shown in Table 4. The Kirollos scale (Table 5), VAS, muscle strength, and JOA scores were used to evaluate the surgical effects of patients in the early and long-term postoperative follow-up assessments, and the analysis results are shown in Table 6.

**Table 3:** The number of appearance of the tether-causing factor.

Tether-causing factor	The number of times
Filum terminal type	120
Arachnoid type	112
Cele type	78
Lipoma type	65
Bone type	34
Diastomyelia type	27

**Table 4:** Individualized treatment strategies for different tether-causing factors in patients with complex tethered cord syndrome.

	Filum terminal type	Arachnoid type	Lipoma type	Bone type	Diastomyelia type	Cele type
Imaging findings	Thickening/lipomatosis	Structural disorder	Lipoma signal	Paramorphia	Double spinal cord	Lipomyelomeni-ngocele or meningocele
Intraoperative findings	High tension	Adhesion, wrap	Surrounding the spinal cord	Spine malformatio-n	Bone or frenulum	Extraspinal
Surgical strategy	Division	Peel	Excision	Release entrapment	Excision partition	Return the spinal canal
Findings needing attention	Distingui-shing boundary	Thinning spinal cord similar to arachnoid	Block the neural substrate	Ensure the stability of the spine	Repair dural sac	Recovery structure
Expected effect	Terminal ionizatio-n	Free the spinal cord	Total or subtotal cut	Perfect spinal canal	Relieve pressure	Return the contents

**Table 5:** Evaluation of the degree of tethered cord release in the 4 groups with the kirollos scale.

	A group	B group	C group	D group	Total
Grade 1	67 (94.4%)	32 (86.5%)	9 (81.8%)	4 (57.1%)	112 (88.9%)
Grade 2	4 (5.6%)	5 (13.5%)	2 (18.2%)	2 (28.6%)	13 (10.3%)
Grade 3	0	0	0	1 (14.3%)	1 (0.8%)
Total	71	37	11	7	126

88.9% of patients had the degree of surgical release reaching grade 1. Group A > Group B > Group C > Group D, indicating that the more tether-causing factors, the lower proportion of patients reaching grade 1. Among the 4 groups, only 1 case in group D failed to release.

**Table 6:** Evaluation of surgical results in patients with complex tethered cord syndrome.

	Early postoperative period			Long-term follow-up		
	Improved	Stable	Aggravated	Improved	Stable	Aggravated
A (71)	49 (69%)	18 (25.4%)	4 (5.6%)	60 (84.5%)	10 (14.1%)	1 (1.4%)
B (37)	23 (62.2%)	8 (21.6%)	6 (16.2%)	30 (81.1%)	5 (13.5%)	2 (5.4%)
C (11)	6 (54.5%)	3 (27.3%)	2 (18.2%)	8 (72.7%)	2 (18.2%)	1 (9.1%)
D (7)	3 (42.9%)	3 (42.9%)	1 (14.2%)	4 (57.1%)	2 (28.6%)	1 (14.3%)
Total	81 (64.3%)	32 (25.4%)	13 (10.3%)	102 (80.9%)	19 (15.1%)	5 (4%)

The analysis of long-term follow-up results showed that the improvement rate of Group A > Group B > Group C > Group D.

Patients with more tether-causing factors generally show more complicated conditions, greater surgical difficulty, greater surgical risk, and a higher incidence of postoperative complications. On the basis of the research data, the results of early and long-term postoperative follow-up also conform to this law. Long-term follow-up after rehabilitation treatment indicated that most patients showed improvement in symptoms, including better recovery of patients who showed improvement in the early postoperative period, improvement of postoperative stable patients in comparison with preoperative patients, and recovery of patients showing disease aggravation to preoperative levels.

### 3.2 Analysis of typical cases

#### 3.2.1 Case 1

**Figure 1:**
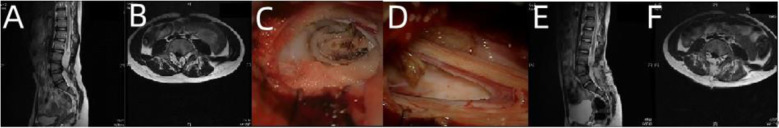
Imaging examination and intraoperative findings of case 1 before and after operation. (**A**,**B**) Preoperative MRI T2-weighted images of the lumbosacral vertebrae (sagittal and axial) showed diastematosis caused by a bone ridge. (**C**,**D**) Image obtained after the bone ridge was exposed and excised during the operation; (**E**,**F**) postoperative reexamination showed that the bone ridge was excised and the spinal cord was released satisfactorily.

The patient in this case was a 9-year-old girl showing a lumbosacral mass with bipedal deformity for 9 years and abnormal urination for 8 years who was admitted to the hospital on June 13, 2016. The patient had shown varus deformity since childhood and underwent orthopedic surgery several times. After an early improvement, the varus deformity appeared again. Since childhood, she had been passing stool 3–4 times/day and showed intermittent defecation difficulties, dysuria, weakness in urination and dripping. Physical and neurological examinations showed a lumbosacral mass approximately 0.5 cm in diameter with hair distribution on the surface. Sphincter ani slacked. The patient showed scoliosis, bipedal varus deformity, ankle joint stiffness, poor motion, a heavier right foot, decreased shallow sensation in both lower limbs, especially the heavier foot, level IV muscle strength in the right lower limb, and level IV + muscle strength in the left lower limb. The patient was diagnosed as showing (1) TCS, (2) lumbosacral spina bifida, (3) diastematosis of the spinal cord, (4) meningocele, and (5) scoliosis. The tether-causing factors were categorized under the filum terminal type, cele type, and diastomyelia type. The surgical treatment included bone ridge excision, terminalis disconnection, and dural repair to achieve cord tether release (Figure 1).

#### 3.2.2 Case 2

**Figure 2:**
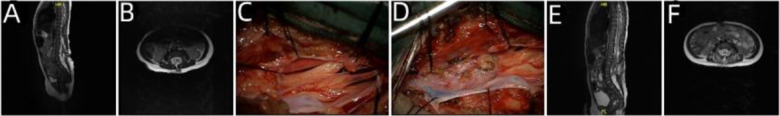
Imaging examination and intraoperative findings in case 2 before and after operation. (**A**,**B**) Preoperative lumbosacral MRI T2-weighted images (sagittal and axial) showed lipomyelomeningocele. (**C**) Intraoperative double filaments; (**D**) the lumen after lipoma resection to avoid postoperative adhesion; (**E**,**F**) postoperative reexamination showed that the spinal cord was restored into the spinal canal, the dura was repaired intact, and the spinal cord was released satisfactorily.

The patient was a 4-month-old girl who was admitted to the hospital on December 3, 2017 due to progressive enlargement of the lumbar depression for 4 months after birth. At birth, the child was found to have a sunken waist with a diameter of approximately 5 mm and a depth of approximately 0.2 mm, which gradually expanded to a diameter of about 6 mm with a dotted red rash around it. The patient passed soft stools once every 3–4 days. Physical signs and neurological examination showed a skin depression with a diameter of approximately 6 mm and a depth of approximately 0.2 mm in the waist with a dotted red rash around it. The anus reflexes disappeared on anus relaxation. Limb movement was not abnormal. The diagnosis at admission was TCS with lipomyelomeningocele and congenital spina bifida. The tether-causing factors were categorized under the filum terminal type (double filament), cele type, lipoma type, and arachnoid type. The lipomyelomeningocele was released and returned spinal cord and nerves into the spinal canal in the operation. The lipoma underwent subtotal excision and decompression, and the final filament was cut and the nerve substrate was closed to ensure complete release of the tetracheal cord (Figure 2).

#### 3.2.3 Case 3

**Figure 3:**
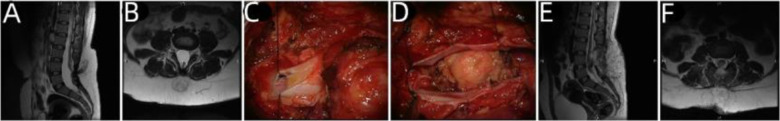
Imaging examination and intraoperative findings before and after the operation in case 3. (**A**,**B**) Preoperative lumbosacral MRI T2-weighted images (sagittal and axial) showed lipomyelomeningocele. (**C**) Intraoperative evidence of tissue swelling and lipoma penetrating inside and outside the dura. (**D**) The spinal cord returned to the spinal canal after lipoma resection. (**E**,**F**) Postoperative reexamination showed that the spinal cord was restored into the spinal canal, the dura was repaired intact, and the spinal cord was released satisfactorily.

The patient was a 36-year-old female who had been experiencing constipation for 34 years and had undergone bipedal deformity surgery 20 years previously who was admitted to the hospital on March 8, 2017 due to weakness of both lower limbs, progressive aggravation of intermittent urinary incontinence for more than 2 years, and significant aggravation for more than 2 months. The patient had been diagnosed as showing a lumbosacral mass at birth, which was resected at the local hospital. She was constipated from childhood, with bowel movements occurring 6–7 days apart. Twenty years before admission, the patient had undergone several procedures for correction of bipedal varus deformity, and her left foot and left ankle were immobile after surgery. Two years before admission, she developed weakness of both lower extremities and progressive aggravation, along with obvious aggravation of the left lower extremity and urinary incontinence with incomplete dripping. Two months before admission, the weakness of the left lower limb worsened significantly, her walking became unstable, her urinary incontinence worsened, and she showed nocturnal enuresis. Physical signs and neurological examination showed surgical scars of approximately 10 cm in length in the lumbosacral region, transverse surgical scars of approximately 6 cm in length in the right heel, and three longitudinal scars of approximately 6 cm in length in the left ankle and left calf. The patient also showed hypoesthesia of the left lower limb, right calf, right foot, and saddle area; bipedal varus deformity, with a more serious deformity in the left foot; grade II muscle strength of the right toe and right ankle and grade IV + muscle strength of the right thigh and right calf; and level 0 muscle strength of the left ankle and left toe and level III + muscle strength of the left thigh and calf muscle. The diagnosis at admission was as follows: (1) TCS; (2) lipomyelomeningocele; (3) spinal lipoma; and (4) congenital spina bifida. The tether-causing factors of the patients were categorized under the cele, lipoma, and arachnoid types. After lipomyelomeningocele release, spinal canal restoration, lipoma subtotal resection, and arachnoid adhesion release were performed to achieve complete release of the tethered cord and spinal canal decompression (Figure 3).

## 4 Discussion

The characteristics of C-TCS include (1) closely adhered spinal cord and nerve roots that are closely wrapped by arachnoid, lipomatous, and other tissues (11–13); (2) complex lumbosacral vertebral deformities, especially excessive lordosis, kyphosis, torsion deformation, or severe spinal canal stenosis, which make the anatomical structure more complex and preclude distinction of the normal spinal cord and nerve roots, thus greatly increasing the difficulty of surgery (10, 14); and (3) mixed growth of lipomas and spinal cord lipomyelomeningocele or meningingocele and difficulty in decompression of spinal canal contents (8). The imaging findings of patients with C-TCS are more varied and complex than those of patients with common tethered cord syndrome, and the factors causing the tethered cord are complex and sometimes even difficult to identify in C-TCS, necessitating a different choice of surgical options (7, 15). Individualized surgery is more important in the treatment of patients with C-TCS (16–19).

The main objectives of tethered cord release are release and decompression (20, 21) to reduce the spinal cord tension and thereby reduce the rate of retethering (22, 23). C-TCS is called “complex” because these tether-causing factors increase the difficulty of release and decompression, with the filum terminal, arachnoid, bone, and diastomyelia types showing greater difficulty of release and the lipoma and cele types showing greater difficulty of decompression (24–26). Since complete release and full decompression are the primary objectives of tethered cord release (27), an understanding of the tether-causing factors responsible for C-TCS is essential (28, 29). The main factor causing a tethered cord of the filum filament type is the filament, while the secondary factors include lipomas and lipomyelomeningocele/meningocele (30, 31). The arachnoid type is primarily caused by arachnoid adhesion, while the secondary factors include lipoma and lipopomyelomeningocele/meningocele (32, 33). The factors responsible for the cele type include lipomyelomeningocele/meningocele, and the secondary factors include lipoma, spina bifida, and arachnoid adhesion. The primary factor responsible for the lipoma type is lipoma, and the secondary factors include lipomyelomeningocele/meningocele and arachnoid adhesion (34, 35). The primary factor responsible for the bone type is spinal malformation, while secondary factors include thickening of filaments, arachnoid adhesion, and lipomyelomeningocele/meningocele. The primary factor responsible for the diastematosis type is diastematosis of the spinal cord (type I and II), and the secondary factors include spina bifida and arachnoid adhesion (36, 37). Thus, C-TCS is not characterized by the so-called isolated thrombolytic factors and is mediated by the joint action of multiple tether-causing factors. For this reason, accurate differentiation of the responsible tether-causing factors is of great guiding significance for surgery. However, complete release of the tethered cord can only be achieved if all tether-causing factors are addressed, regardless of whether they are primary causative factors or secondary factors (38, 39).

The severity of deformity in TCS has been shown to be directly related to the risk of surgery, and quantifying the complexity of TCS has been a topic of great interest and a difficult problem (40). The causes showing spinal low position, poor spinal motion and spinal cord compression have been analyzed, classified, and summarized into six general tether-causing factors. Using this approach, the complexity of the patient's condition can be preliminarily assessed by analyzing the number of tether-causing factors in each patient (41, 42). In this study, data analysis of 126 patients with C-TCS showed that the more tether-causing factors, the more complicated the condition of patients, the more difficult the operation, the greater the risk of surgery, and the higher the incidence of postoperative complications. In addition, patients with the same number of tether-causing factors also show large differences in performance, so individualized treatment is an important topic that requires further research (43).

The surgical strategies differed according to the factors shown by the patient: (1) the filum filament type required complete disconnection of the filum filament tissue (especially the inner filum filament, since simply disconnecting the outer filum filament cannot achieve the purpose of teaming release), pay more attention to the complete disconnection of the double filum filament to avoid omissions (44). (2) The arachnoid type required detachment of the arachnoid adhesion, which involved first distinguishing the spinal cord, nerves, and hyperplasia of the arachnoid and simultaneous detachment and release of the end of the spinal cord and nerve adhesion to avoid damage to the spinal cord and nerves (45). It was better to distinguish the security interface. (3) In the lipoma type, the spinal cord lipoma usually wraps the end spinal cord, and to avoid damage to the spinal cord and nerves wrapped in the lipoma during the resection process, the lipoma can be removed as much as possible to achieve sufficient decompression while maintaining safety; a small amount of lipoma can be retained when necessary, but the lipoma must be thoroughly stripped of the adhesion to the surrounding tissue, and the exposed nerve substrate must be sutured to closure. By enlarging the space, reducing the volume of lipoma and closing the wound, the retethering rate was reduced (46, 47). (4) For the bone type, to ensure the stability of the spine, the deformed bone caused by compression should be removed or ground as far as possible, with the purpose of relieving nerve compression, expanding free nerve activity range, reducing retethering rate, and the first or second stages of spinal internal fixation surgery should be performed if necessary (36). (5) In the diastematosis type, the factors causing diastematosis mainly include malformed bone, fibrocartilage, or fibrous frenulum. The diastematosis is usually located above the tethered cord, which should be accurately positioned to avoid excessively long surgical incision. Although the spinal cord showing diastematosis cannot be recovered, the release of spinal cord compression can reduce the tension of the spinal cord and relieve symptoms, at the same time, the integrity of the dura was restored (48). (6) In the cele type, which is often accompanied by spina bifida, most cases show backward bulging, while a few show forward bulging. The bulging spinal cord is completely separated from the adherent subcutaneous tissue and muscle; the spinal cord and nerves are returned to the spinal canal; and the dura is closely repaired, enlarged and repaired if necessary. Advancements in material technology have resulted in ongoing optimization of the biological stability, biosafety and biocompatibility of spinal replacement materials. Thus, an increasing number of patients are choosing to undergo spina bifida repair, restore the integrity of the spinal canal, maintain the stability of the pressure in the spinal canal, and increase the stability of the spine (49).

C-TCS is usually caused by three or more tether-causing factors. Individualized therapy employing different surgical strategies for different patients is important to finally achieve the purpose of complete release of tethered cord (50, 51). For example, in the filum filament + diastematosis type, the location of filament release is located at S1–2, or even lower, and the diastematosis is located at L1–2, which requires two surgical incisions to address both of these factors and achieve the surgical objective. For patients with C-TCS, individualized surgical treatment is aimed at completely releasing the tether while protecting the terminal spinal cord and nerves as much as possible, thereby preventing or delaying the aggravation of neurological dysfunction (52). The number of patients included in this study was slightly insufficient for an in-depth assessment of C-TCS, and data from more cases are needed. Moreover, patients show varying degrees of neurodevelopmental malformation, and in addition to continuing to study the quantitative criteria for TCS severity, the possibility of quantifying individualized treatment is also a topic for further research (53). In addition, the role of each tether-causing factor in spinal cord and nerve injury also needs to be further studied.

## Data Availability

The original contributions presented in the study are included in the article/Supplementary Material, further inquiries can be directed to the corresponding authors.
